# Synthesis and Characterization of Gd_2_O_3_ Hollow Microspheres Using a Template-Directed Method

**DOI:** 10.3390/ma9050323

**Published:** 2016-04-28

**Authors:** Xueliang Jiang, Lu Yu, Chu Yao, Fuqing Zhang, Jiao Zhang, Chenjian Li

**Affiliations:** College of Materials Science and Engineering, Wuhan Institute of Technology, Wuhan 430074, Hubei, China; 201301041@wit.edu.cn (L.Y.); 04002085@wit.edu.cn (F.Z.); zhangjiao@wit.edu.cn (J.Z.); leechenjian@wit.edu.cn (C.L.)

**Keywords:** gadolinium oxide, hollow microspheres, preparation, photooxidation

## Abstract

Uniform rare-earth gadolinium oxide (Gd_2_O_3_) hollow microspheres, as formed through a urea-assisted homogenous precipitation process using carbon spheres as a template and a subsequent heat treatment, were characterized by using X-ray diffraction, Fourier transformed infared spectroscopy, thermogravimetry, X-ray photoelectron spectroscopy, scanning electron microscopy, transmission electron microscopy and Brunauer-Emmett-Tellet surface area measurement. The results indicate that the final products can be indexed to a cubic Gd_2_O_3_ phase with high purity and have a uniform morphology at 500 nm in diameter and 20 nm in shell thickness. The as-synthesized Gd_2_O_3_ hollow microspheres exhibited a superior photooxidation activity to that of Gd_2_O_3_ powder and an effect similar to P25, significantly broadening the potential of Gd_2_O_3_ hollow microspheres for many practical applications.

## 1. Introduction

Monodispersed hollow nano/microspheres of uniform size and shape have attracted considerable research interest owing to their low density, large specific surface area, good surface permeability properties, and a wide range of potential applications such as catalysis, drug delivery, chemical sensors, lithium-ion batteries, *etc.* [1,2,3,4,5,6,7]. Recently, many efforts have been made in the development of different methods for design and preparation of hollow microspheres materials. Among these methods, the most developed seem sto be the template method, including hard templates (such as carbon spheres [8], silica particles [4], melamine formaldehyde (MF) microspheres [5]) and soft templates (such as liquid droplets, vesicles, emulsion, gas bubbles) [9,10,11]. However, these methods have some inherent drawbacks in template formation and removal. Usually tedious multistep procedures were needed in the template formation and harsh synthetic conditions were required, including the use of volatile organic solvents and non-green etching agents in the template removal. Therefore, the carbon spheres template is considered a good template with the advantages of low cost, mild conditions, easy operation and environmental friendliness.

Rare earth oxides materials have been investigated extensively for their potential applications in luminescent devices, fluorescent labels, medical diagnostics, catalysts and so forth [12]. These potential applications are due to special optical, electronic, and chemical properties arising from their 4f sub-shell electrons [13,14]. Gd_2_O_3_ is a typical functional rare earth oxide, due to significant characteristic properties such as chemical durability, thermal stability, large band gap, high refractive index and high dielectric constant [15,16,17]. Up to now, various morphologies of different sizes, dimensions, and structures such as nanoparticles, nanotubes, curved rods, spheres, plate of Gd_2_O_3_ have been synthesized by a variety of approaches [12]. Boopathi *et al.* reported an inexpensive preparation method for obtaining Er5%: Gd_2_O_3_ nanoscale rods through a co-precipitation process [18]. Guang *et al.* have prepared Gd_2_O_3_ and Eu^3+^-doped Gd_2_O_3_ nanotubes via a simple wet-chemical route at ambient pressure and low temperature followed by a direct annealing process in air [19]. Maria *et al.* obtained the Gd_2_O_3_ nanoparticles via the polyol method using silanes as the innermost stabilizer layer and an outer layer of bifunctional polyethylene glycol (PEG) [20]. As is known, Gd_2_O_3_ has been widely applied in luminescence, MRI contrast imaging, drug delivery and so on [17]. Gd_2_O_3_ hollow microspheres were combined with the special properties of Gd_2_O_3_ and the structural effects of hollow microspheres, while their applications for optical, electronic, magnetic and catalytic properties were greatly extended [14]. However, the photocatalysis oxidation property of Gd_2_O_3_ hollow microspheres has received less attention.

Herein we developed an easy and rapid method to obtain monodisperse Gd_2_O_3_ hollow microspheres through a urea-assisted homogenous precipitation process using carbon spheres as templates and a subsequent heat treatment. The synthesis is performed in an aqueous solution without using any organic template and a further etching process, which usually uses an acid or base as an etching agent. Finally, the structure, morphology, formation process, and photooxidation performance of the Gd_2_O_3_ hollow microspheres are characterized in detail.

## 2. Experimental Section

### 2.1. Materials

Gd(NO_3_)_3_·6H_2_O (99.9%), glucose, urea, formaldehyde, Ethanol absolute (EtOH) were analytical grade reagents and used directly without further purification. All materials were supplied by Sinopharm Chemical Reagent Co., Ltd. (Shanghai, China) except Gd(NO_3_)_3_·6H_2_O (99.9%) was purchased from Aladin (Shanghai, China). In addition, deionized water was made in our laboratory.

### 2.2. Preparation of Monodisperse Carbon Spheres

The carbon spheres templates were prepared through the poly condensation reaction of glucose under hydrothermal conditions in a Teflon-lined stainless steel autoclave. In a typical procedure, glucose (2 g) and formaldehyde (2 mL) were dissolved in 50 mL of deionized water to form a clear solution. The solution was then sealed in a 100 mL Teflon-lined stainless autoclave and maintained at 160 °C for 6 h. After the autoclave was naturally cooled to room temperature, the black-brown precipitates were obtained by centrifugation, washed three times with ethanol and deionized water and then dried at 80 °C for 6 h in air.

### 2.3. Synthesis of Gd_2_O_3_ Hollow Microspheres

In a typical synthesis, 2 mL of 0.5 M Gd(NO_3_)_3_ aqueous solution was added to 30 mL of deionized water. Then, the 3.0 g of urea was dissolved in the solution to form a clear solution by vigorous stirring. Subsequently, 0.1 g of the as-prepared carbon spheres was added and dispersed into the above solution with the assistance of ultrasonication for 15 min. Then, the mixture was transferred to a round-bottomed flask and heated to 85 °C for 6 h with vigorous stirring. Finally, the precursor was collected by centrifugation and washed with deionized water and ethanol several times and dried in air at 60 °C for 8 h. The final hollow Gd_2_O_3_ microspheres were obtained through a calcination process at 800 °C in air for 2 h.

### 2.4. Characterization of Gd_2_O_3_ Hollow Microspheres

The crystal structure of the samples was characterized by X-ray diffraction (XRD, XD-5A, Shimadzu, Kyoto, Japan) using Cu Kα radiation. The morphology and size of the samples were studied by scanning electron microscopy (SEM, JEM-5510LV, JEOL, Tokyo, Japan) and transmission electron microscopy (TEM, JEM-2100, JEOL, Tokyo, Japan). The chemical composition of the samples was investigated by X-ray photoelectron spectroscopy (XPS, AXIS-ULTRA DLD-600W, Shimadzu-Kratos, Kyoto, Japan) using Al Kα as the X-ray source. Thermogravimetry (TG,) curves of the samples was carried out with a Netzsch STA 409 PC analyzer (Netzsch, Selb, Bavaria, Germany) at a heating rate of 10 °C/min from 25 to 800 °C. Fourier transformed infared spectroscopy (FT-IR) was measured by a KBr pellet technique and spectra of the powders recorded under vacuum conditions using a Thermo Electron Nicolet-6700 spetrometer (Thermo Electron Nicolet, Madison, WI, USA). Nitrogen adsorption-desorption analysis was performed with a NOVA 2000e (Quantachrome, Boynton Beach, FL, USA), and the specific surface area was determined by the Brunauer-Emmett-Telletr (BET) method. Elemental analysis (EA) was carried out using a vario EL cube 19093019 (Elementar, Frankfurt Hanau, Hessen, Germany). UV-visible absorption spectra were recorded on PerkinElmer Lambda 35 spectrophotometer (PerkinElmer, Fremont, CA, USA) equipped with an integrating speres. All the measurements were carried out at room temperature.

### 2.5. Photocatalytic Activity Measurement

The photocatalytic activity of catalysts was evaluated by the degradation of methyl orange (MO) in an aqueous containing 0.06 M MO solution, 0.1 g catalysts and 5 mL hydrogen peroxide solution in 50 mL glass vessels by referring previous publications [21]. A halogen tungsten lamp was used as simulated solar light irradiation (350 W). The 420 nm cut off filter was used to get the visible light source. The suspension was stirred vigorously for 30 min in the dark to establish adsorption-desorption equilibrium of MO, and the degradation reaction started under simulated solar light irradiation with constant magnetic stirring. Samples were withdrawn periodically from the reactor, then centrifuged and analyzed by recording variations in the absorption in the Ultraviolet–visible (UV-vis) spectra of MO (λ = 464 nm). The percentage degradation (η) of MO was calculated with the following formula [22].

η = [(*A*_0_ − *A*_t_)/*A*_0_] × 100% (1)

Here, *A*_0_ is the initial absorbance of MO solution when the samples established adsorption-desorption equilibrium in MO solution, *A*_t_ is the absorbance of MO solution on a reaction time *t*.

## 3. Results and Discussion

### 3.1. XRD Analysis

**Figure 1:**
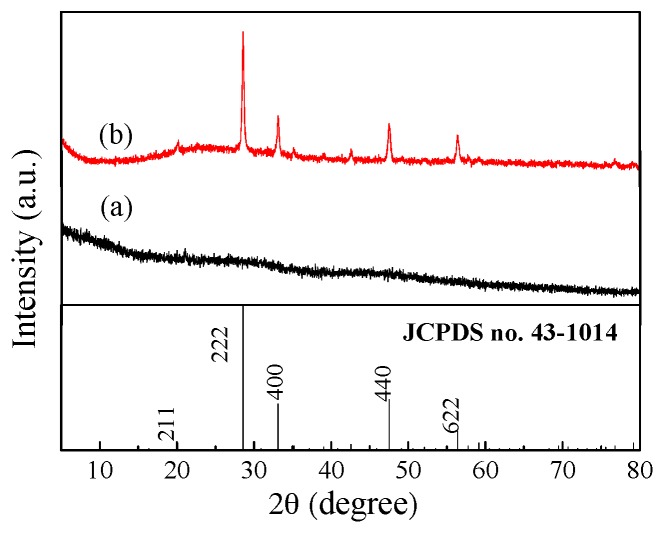
XRD patterns of the samples for (**a**) uncalcined precursor and (**b**) after calcination at 800 °C for 2 h. The standard data for cubic-phase Gd_2_O_3_ (JCPDS No. 43-1014) is presented for comparison.

XRD patterns of the uncalcined precursor and Gd_2_O_3_ hollow microspheres after calcination at 800 °C for 2 h are shown in Figure 1. No obvious diffraction peaks are detected and the amorphous structures are defined for the uncalcined precursor (Figure 1a). After 800 °C calcinations, all the diffraction peaks of the hollow microspheres (Figure 1b) can be well indexed to pure cubic Gd_2_O_3_ phase corresponding to the standard card (JCPDS No. 43-1014). Furthermore, no additional peaks have been found, indicating the formation of a purely cubic Gd_2_O_3_ phase. The strong and sharp diffraction peaks indicate the as-obtained sample is well crystallized. Hence, it can be inferred that the calcination process has a dual function: the formation of hollow crystalline structures from the amorphous precursor layer and elimination of carbon sphere cores.

### 3.2. FT-IR Analysis

**Figure 2:**
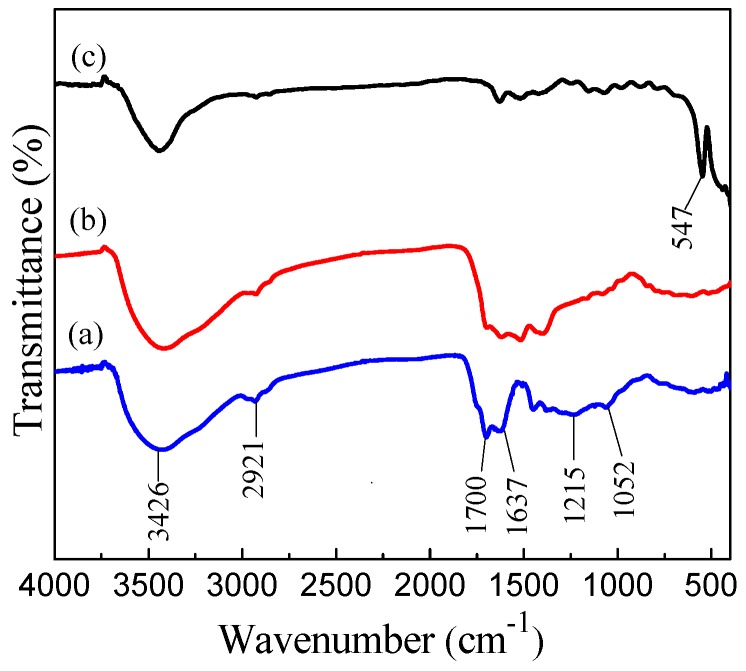
FT-IR spectra of (**a**) carbon spheres; (**b**) uncalcined precursor; (**c**) Gd_2_O_3_ hollow microspheres.

FT-IR spectra were used to investigate the functional groups of the carbon spheres, uncalcined precursor and Gd_2_O_3_ hollow microspheres, as shown in Figure 2. The FT-IR spectrum of the carbon spheres (Figure 2a) shows the absorption bands, which are located at about 3426, 2921, 1700, 1637, 1215, 1052 cm^−1^ and assigned to the vibrations of hydroxyl (–OH), alkane hydrocarbon (C–H), carbonyl (C=O), unsaturated C=C, C–OH and glycosidic –C–O–C– linkage, respectively. The presence of glycosidic –C–O–C– linkage confirms the polymerization reaction of glucose, and the unsaturated C=C groups indicate that a carbonization process has occurred during the formation process of carbon spheres [23,24]. The –OH, C=O on the surface improve the dispersibility and stability of the carbon spheres in aqueous solution. Moreover, the result of EA shows that the as-prepared carbon spheres contain 62.6 wt % of C, 5.063 wt % of H and 32.337 wt % of O, the atomic ratio (C:H:O) is 5:5:2. The FT-IR spectrum of the uncalcined precursor (Figure 2b) is similar to that of the carbon spheres, but weaker in intensity. Figure 2c shows the FT-IR spectrum of the hollow Gd_2_O_3_ microspheres. It can be see that all of the functional groups of the carbon spheres and the precursor nearly disappear, indicating the carbon spheres have been completely removed and the transformation from the precursor to the hollow structure product has occurred. Moreover, a new band at 547 cm^−1^ appears and can be assigned to the Gd–O stretching frequencies of Gd_2_O_3_ [25,26]. The result is consistent with that of XRD pattern and confirms the formation of crystalline Gd_2_O_3_ hollow microspheres via urea-based homogeneous precipitation and the further calcination.

### 3.3. TG Analysis

**Figure 3:**
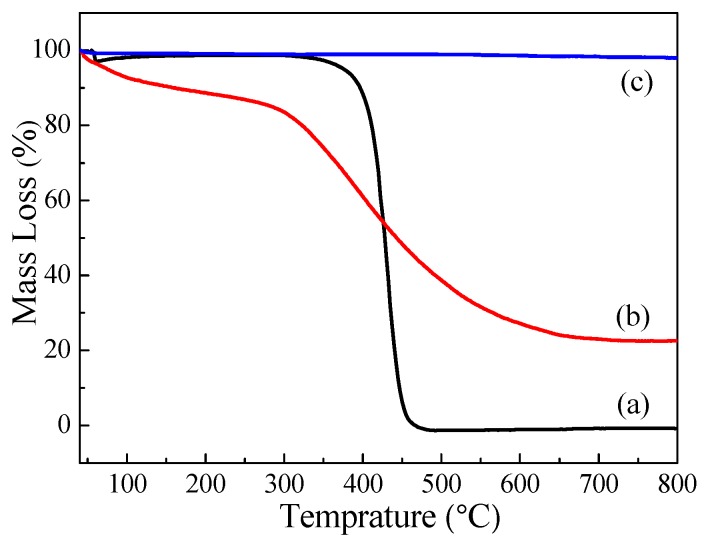
TG curves of (**a**) carbon spheres; (**b**) uncalcined precursor; (**c**) Gd_2_O_3_ hollow microspheres.

The thermal behaviors of the carbon spheres, uncalcined precursor and Gd_2_O_3_ hollow microspheres are investigated by TG measurements in Figure 3. It can be seen from the carbon spheres template (Figure 3a), the weight loss of the template is nearly 100% when the temperature is elevated to 500 °C, revealing that the carbon sphere template was completely removed after calcinations. Three steps of weight loss can be discovered in the TG curves of uncalcined precursor (Figure 3b). The first slow weight loss can be associated with the dehydration and densification of the carbon spheres. The second sharp weight loss may be attributed to the burning of the carbon spheres template between 300 and 600 °C. Beyond 600 °C, the mass of the uncalcined precursor still slowly decreases until 700 °C, which can be assigned to the decomposition of the precursor to crystalline Gd_2_O_3_. We can see from the TG curve of Gd_2_O_3_ hollow microspheres (Figure 3c) there is almost no weight loss can be observed. This demonstrates that the final product is pure Gd_2_O_3_, a result in accordancd with the XRD pattern.

### 3.4. XPS Analysis

**Figure 4:**
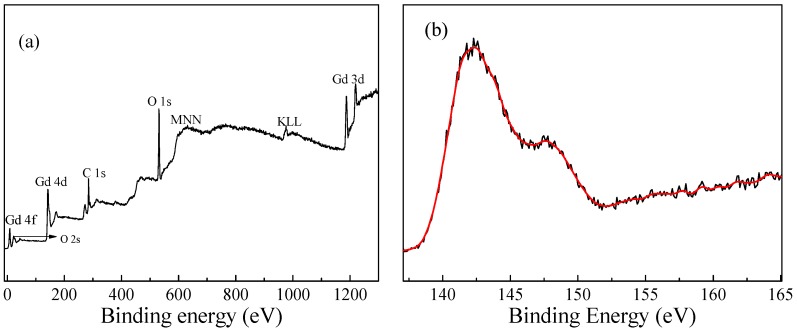
XPS patterns of Gd_2_O_3_ hollow microspheres: (**a**) XPS wide spectrum; (**b**) Gd 4d spectrum.

The chemical composition of the Gd_2_O_3_ hollow microspheres was investigated by XPS in Figure 4. Figure 4a represents the survey XPS spectrum, wherein all the peaks can be assigned to electronic transitions in Gd, O, and C. The high-resolution XPS spectrum of Gd 4d is shown in Figure 4b. The two strong peaks at 142.28 eV and 147.48 eV corresponding to a spin-orbit splitting of 5 eV corresponding respectively to the 4 d_5/2_ and 4 d_3/2_ energy levels of Gd. These observation are in good agreement with reports in the literature for Gd_2_O_3_ [17,27]. The peaks around 531.08 eV (Figure 4a) can be assigned to the bond between O^2−^ and Gd^3+^ [28]. From the above result, the pure Gd_2_O_3_ hollow microspheres were obtained successfully.

### 3.5. Morphology and Microstructure

**Figure 5:**
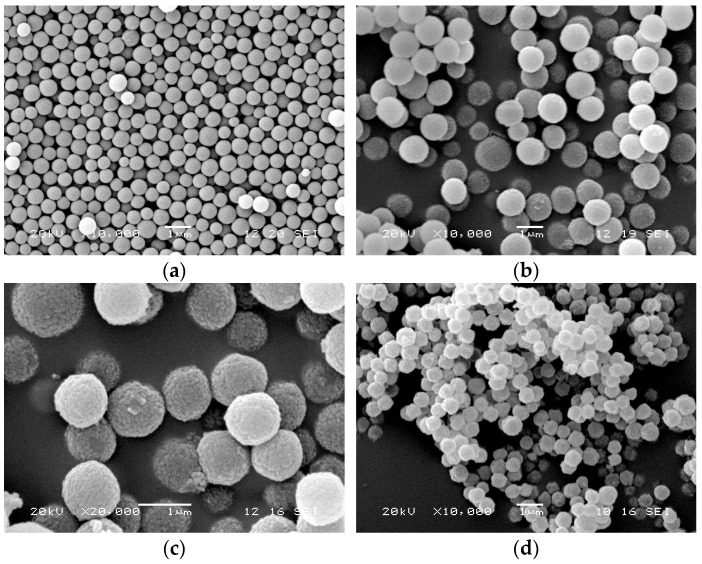
SEM images of (**a**) carbon spheres; (**b**,**c**) uncalcined precursor; (**d**) Gd_2_O_3_ hollow microspheres.

**Figure 6:**
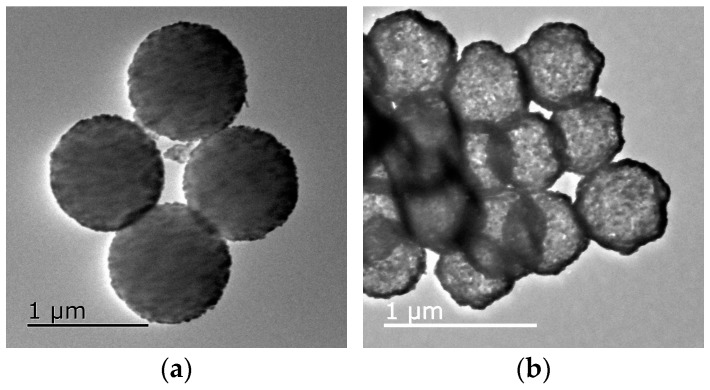
TEM images of (**a**) uncalcined precursor; (**b**) Gd_2_O_3_ hollow microspheres.

The morphology and microstructure of carbon spheres, uncalcined precursor and Gd_2_O_3_ hollow microspheres were analyzed by SEM and TEM technique, as shown in Figure 5 and Figure 6. Figure 5a shows the SEM image of the as-prepared carbon spheres. It can be seen that the carbon spheres template consists of well-dispersed microspheres with a smooth surface and a narrow size distribution in the range, with an average diameter of approximately 600 nm. Figure 5b shows the SEM image of the uncalcined precursor. It is noted that the spherical morphology and good dispersion of the uncalcined precursor remained but the surface is much rougher (Figure 5c) and the particle size is slightly higher than that of the rare carbon spheres due to the formation of an amorphous shell that was composed of a large number of uniform nanoparticles. Figure 5d shows the SEM images of the sample after calcination at 800 °C for 2 h. The obtained sample consists of a large amount of uniform, well-dispersed Gd_2_O_3_ hollow microspheres which are similar to the precursor, which implies that the carbon spheres templates essentially determine the shape and the size of the final products. In addition, it can be seen that the average diameter of the Gd_2_O_3_ hollow microspheres around 500 nm decrease in comparison with the uncalcined precursor, which may be assigned to the dehydration of the cross-linked structure of the carbon spheres templates and the densification of the precursor on the surface layer when the amorphous Gd(OH)CO_3_ shell converted to the closely compact Gd_2_O_3_ phase during the calcination process. The TEM images of the uncalcined precursor (Figure 6a) show spherical morphology with well-dispersed and solid structure. The TEM images of the sample after calcination at 800 °C for 2 h (Figure 6b) reveal the hollow nature of the Gd_2_O_3_ microspheres through the strong contrast between the dark edge and the pale center, and the shell thickness is about 20 nm.

### 3.6. BET Surface Area Analysis

**Figure 7:**
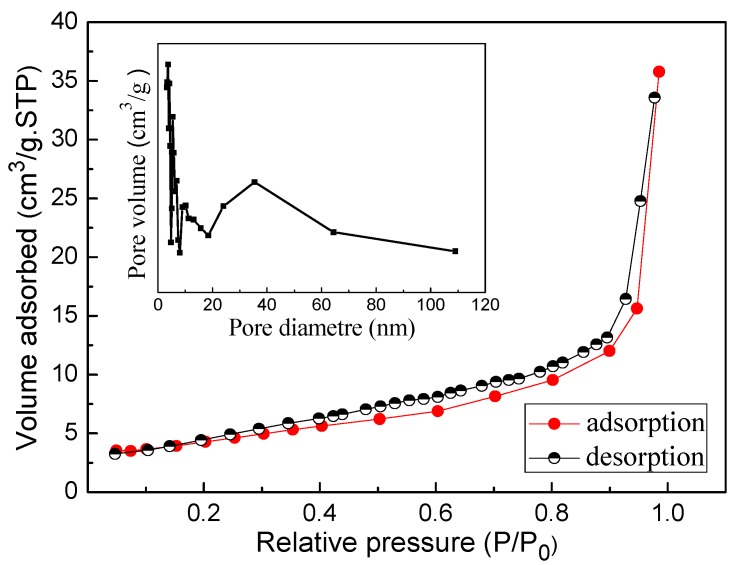
N_2_ absorption-desorption isotherms of Gd_2_O_3_ hollow microspheres. Inset: pore size distribution of Gd_2_O_3_ hollow microspheres.

Figure 7 shows the N_2_ adsorption-desorption isotherm and pore size distribution of the Gd_2_O_3_ hollow microspheres. The Brunauer-Emmett-Telletr (BET) surface area is 15.2 m^2^/g, and the pore volume is 0.051 cm^3^/g, which is not particularly large since the material is predominantly macroporous with thin shells. The pore size distribution is centred at 3.85 nm as calculated using the Barrett-Joyner-Halenda (BJH) method from the absorption branch (inset in Figure 7). The pores are possibly attributable to the interstitial spaces between nanoparticles in the shell. The result reveals that the Gd_2_O_3_ hollow microspheres have a mesoporous pore shell, which is beneficial to light harvesting. Table 1 summarizes the results of different samples calculated based on N_2_ adsorption-desorption isotherms. From Table 1 we can see that the specific surface areas and pore volumes of Gd_2_O_3_ hollow microspheres are greater than those of Gd_2_O_3_ powder.

### 3.7. Proposed Formation Process

**Scheme 1:**
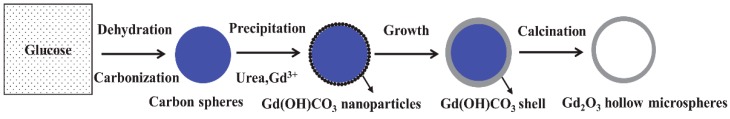
Schematic illustration of the overall formation process of Gd_2_O_3_ hollow microspheres.

As shown in Scheme 1, based on the experimental result and analysis, the possible mechanism for the formation of the Gd_2_O_3_ hollow microspheres can be mainly divided into three stages. Firstly, the carbon spheres templates were formed by the polymerization of glucose molecules and further dehydration of cross-linked polymer by thermal treatment. Secondly, the core-shell structured precursor was obtained by the homogeneous precipitation of the rare earth ions on the surface of the carbon spheres templates using urea as the precipitation agent. FT-IR spectrum confirms the presence of abundant hydroxyl groups on the carbon spheres, which may have a good affinity for Gd^3+^, OH^−^ and CO_3_^2−^ (OH^−^ and CO_3_^2−^ result from the hydrolysis of urea) in aqueous solution. Therefore, the amorphous Gd(OH)CO_3_ nuclei firstly formed in the reaction media may be easily absorbed onto the surface of the carbon spheres. With the reaction proceeding, the nuclei continued to grow and served as the seeds for the growth of Gd(OH)CO_3_ nanoparticles on the surface of the carbon spheres templates, resulting in the core-shell structured composite particles. Finally, the carbon spheres templates were burned out and the amorphous Gd(OH)CO_3_ particles were decomposed into crystalline Gd_2_O_3_ during the calcination process, resulting in the formation of Gd_2_O_3_ hollow microspheres.

### 3.8. Photooxidation Activity of Gd_2_O_3_ Hollow Microspheres

**Figure 8:**
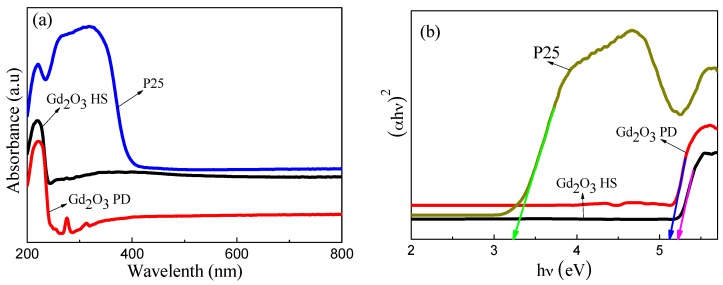
UV-Vis spectrum (**a**) and band gap calculation (**b**) of the different catalysts.

The optical properties of Gd_2_O_3_ hollow microspheres (Gd_2_O_3_ HS), Gd_2_O_3_ powder (Gd_2_O_3_ PD) and P25 (Degussa, Shanghai, China) were examined by UV-visible spectroscopy as shown in Figure 8a. A sharp absorption peak at 220 nm is observed in these three catalysts, two small peaks at 275 nm and 313 nm appeared in Gd_2_O_3_ PD but are not found in Gd_2_O_3_ HS, and a significant increase in the absorption at wavelengths less than 400 nm can be assigned to the intrinsic absorption of P25. The band gap energy of these catalysts could be calculated according to the following formula [18]:

αh*υ* = *A*(h*υ* − *Eg*)*^n^* (2)

where h, *A*, α, *υ*, and *Eg* are the Plank constant, a constant, absorption coefficient, light frequency, and band gap, respectively. *n* is a constant associated with the different types of electronic transitions (for direct band gap allowed transition, *n* = 1/2). The band gap of Gd_2_O_3_ HS, Gd_2_O_3_ PD and P25 were calculated to be 5.23 eV, 5.12 eV and 3.23 eV as shown in Figure 8b. The smaller band gap energy means a wider response range of P25, and the sample can absorb more photons, which would contribute to an enhanced photocatalytic activity for P25 [29]. The large energy gap means a narrow response range less than 230 nm of UV light of Gd_2_O_3_, and the sample can absorb few photons.

**Figure 9:**
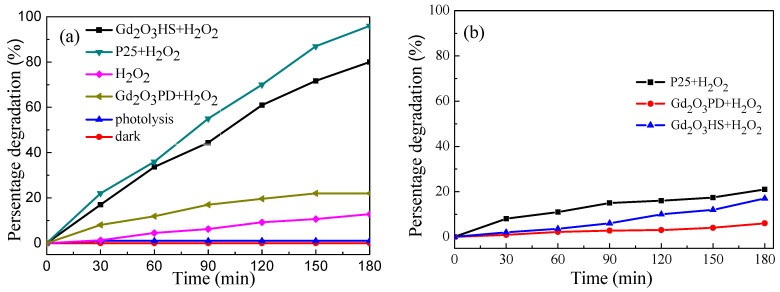
The degradation of MO under (**a**) simulated solar light and (**b**) visible light.

The photo-degradation of MO was investigated under simulated solar light and visible light (λ > 420 nm). Compared with Figure 9a,b, the photo-degradation of MO under visible light for Gd_2_O_3_ hollow microspheres, Gd_2_O_3_ powder and P25 can be seen. Figure 9a shows the degradation of MO under similated solar light for the dark absorption test, photolysis test (no catalyst), H_2_O_2_, Gd_2_O_3_ hollow microspheres, Gd_2_O_3_ powder and P25. As can be seen from the results, the effect of adsorbtion and photolysis of catalyst on the decomposition of MO was negligible during the test period. Single H_2_O_2_ was capable of degrading MO under simulated solar light, because of the formation of hydroxyl radicals via following process. Moreover, for Gd_2_O_3_ hollow microspheres, the photo-degradation of MO is expected not to be promoted if H_2_O_2_ was not added, so the photo-degradation of MO can be regarded as photooxidation.

H_2_O_2_ + h*υ* → 2HO• (3)

It can also be indicated that the photo-degradation effect of MO for Gd_2_O_3_ hollow microspheres was similar to P25, although the photo-degradation mechanism of Gd_2_O_3_ is different from P25 as shown in Figure 8a,b. The photocatalytic activity of P25 toward MO degradation lies in the generation of conduction-band electrons (e^−^) and valence-band holes (h^+^) when irradiated with UV-light existence; the direct oxidation by holes and indirect oxidation by produced hydroxyl radicals are responsible for MO degradation [30,31]. However, for Gd_2_O_3_ hollow microspheres, which can activate H_2_O_2_ molecules to produce hydroxyl radicals [32], the hydroxyl radicals oxidated MO, and the high surface area of Gd_2_O_3_ hollow microspheres is more propitious to the absorption of gaseous products of the initial decomposition. It is also shown that Gd_2_O_3_ HS have higher photooxidation activity than Gd_2_O_3_ PD, which may be due to the increase of the specific surface area and hollow structure of Gd_2_O_3_ hollow spheres.

## 4. Conclusions

Uniform Gd_2_O_3_ hollow microspheres 500 nm in diameter and 20 nm in shell thickness have been successfully prepared by employing carbon spheres as a sacrificial template. The composite precursor was synthesized via urea-based homogenous precipitation on the surface of the carbon spheres. Then, subsequent calcination removed the carbon spheres and converted the amorphous shells to crystalline Gd_2_O_3_, resulting in the formation of Gd_2_O_3_ hollow microspheres. The as-prepared Gd_2_O_3_ hollow microspheres exhibited a superior photooxidation activity for the degradation of MO under simulated solar light irradiation than the Gd_2_O_3_ powder and an effect similar to P25 photocatalysts, significantly broadening their potential for many practical applications.

## Figures and Tables

**Table 1:** Surface area measurement and pore volume of the different samples.

Samples	BET Surface Area (m^2^/g)	Pore Volume (cm^3^/g)
Gd_2_O_3_ hollow microspheres	15.211	0.051
Gd_2_O_3_ powder	1.342	0.004
